# Toward a National Tuberculosis Lateral-Flow Urine Lipoarabinomannan (LF-LAM) External Quality Assessment Program in Thailand: Feasibility Demonstration Through Panel Development, Validation, and Proficiency Testing, March 2025–April 2026

**DOI:** 10.3390/diagnostics16203323

**Published:** 2026-10-13

**Authors:** Supaporn Suparak, Theerawit Tasaneeyapan, Petai Unpol, Sirilada Pimpa Chisholm, Siriphailin Jomjunyoung, Kanokwan Ngueanchanthong, Wipawee Thanyacharern, Nitis Smanthong, Thanapatra Klawech, Phitchamon Thongthirach, Laddawan Thiemsing, Chonthicha Kawidam, Chavachol Setthaudom, Somboon Nookhai, Rangsima Lolekha, Pilailuk Akkapaiboon Okada, Sarawut Boonsuk, Sanny Northbrook

**Affiliations:** 1 Department of Medical Sciences, Ministry of Public Health, Nonthaburi 11000, Thailand; petai.u@dmsc.mail.go.th (P.U.); siriphailin.j@dmsc.mail.go.th (S.J.); kanokwan.n@dmsc.mail.go.th (K.N.); s_nitis@kkumail.com (N.S.); thanapatra.kl@dmsc.mail.go.th (T.K.); phitchamon.t@dmsc.mail.go.th (P.T.); laddawan.t@dmsc.mail.go.th (L.T.); chonthicha.k@dmsc.mail.go.th (C.K.); pilailuk.o@dmsc.mail.go.th (P.A.O.); wutmd39ju@hotmail.com (S.B.); 2 Division of Global HIV and Tuberculosis, U.S. Centers for Disease Control and Prevention, Nonthaburi 11000, Thailand; hqd4@cdc.gov (T.T.); hpz3@cdc.gov (S.N.); hpu8@cdc.gov (R.L.); gdo0@cdc.gov (S.N.); 3 Department of Thai Traditional and Alternative Medicine, Ministry of Public Health, Nonthaburi 11000, Thailand; sirilada.p@dmsc.mail.go.th (S.P.C.); wipawee.th@dtam.mail.go.th (W.T.); 4 Clinical Immunology Laboratory, Department of Pathology, Faculty of Medicine Ramathibodhi Hospital, Mahidol University, Bangkok 10400, Thailand; chavachol@hotmail.com

**Keywords:** tuberculosis, lipoarabinomannan, LF-LAM, external quality assessment, proficiency testing, point-of-care testing, HIV/TB coinfection, advanced HIV disease, Thailand

## Abstract

**Background**: The World Health Organization and the Thailand National HIV Treatment Guidelines recommend the use of lateral flow urine lipoarabinomannan (LF-LAM) testing for tuberculosis (TB) detection in people living with HIV (PLHIV) with advanced HIV disease who have signs and symptoms of TB or are seriously ill. As Thailand decentralizes LF-LAM testing, a robust quality assurance system is needed to maintain diagnostic accuracy across healthcare levels. **Methods**: We developed, validated, and rolled out Thailand’s first national external quality assessment (EQA) and internal quality control (IQC) framework for urinary TB LF-LAM testing. Pre-characterized urine panels were distributed to 20 provinces spanning 12 health regions nationwide across four cycles from 2025 to 2026. Longitudinal monitoring of reporting compliance, pass rates, and failure modes was performed across 30 participating sites in 2025 and 64 and 42 sites in 2026 Rounds 1 and 2, respectively. **Results**: IQC coverage increased from 7 to 68 facilities by the final cycle in January 2026, with satisfaction rates of 85.7–100%. EQA pass rates showed a stepwise improvement: 83.3% in Round 1/2025, 93.1% in Round 2/2025 and 97.5% and 98.4% in the 2026 rounds (*p* < 0.05). Initial failures were primarily due to false-positive interpretation of Grade 1 bands and deviation in the reading window. Targeted retraining and standardized reference match-cards reduced user-dependent false-positive results to 0% in the final cycle. **Conclusions**: A centralized national quality assurance framework may be an effective mechanism for standardizing the reliability and interpretation of point-of-care LF-LAM testing across decentralized healthcare systems. This approach strengthens diagnostic capacity at peripheral facilities and supports the safe expansion of TB services for PLHIV.

## 1. Introduction

Tuberculosis (TB), caused by *Mycobacterium tuberculosis*, remains the leading cause of mortality among people living with HIV (PLHIV) [1]. The intersection of the HIV and TB epidemics presents diagnostic challenges, particularly in individuals with advanced HIV disease (AHD) and severe immunosuppression, typically defined by a CD4 T-lymphocyte count of <200 cells/mm^3^ [2]. In this subpopulation, TB frequently manifests with atypical clinical features, including extrapulmonary involvement and paucibacillary pulmonary disease, drastically reducing the diagnostic yield of conventional sputum-based approaches [3]. Critically ill or severely cachectic patients often cannot produce adequate, high-quality sputum specimens, underscoring an urgent clinical need for non-sputum-based diagnostic alternatives [4].

The World Health Organization (WHO) recommends the lateral flow urine lipoarabinomannan (LF-LAM) assay as an adjunctive point-of-care test to meet this need, for use in hospitalized and ambulatory PLHIV with AHD [5]. Lipoarabinomannan (LAM), a key structural glycolipid of the *M. tuberculosis* cell wall, is cleared through the kidneys and excreted into the urine during disseminated or renal TB infection, making it detectable in a non-invasive, readily obtainable biofluid [6]. Rapid immunochromatographic detection of urinary LF-LAM delivers actionable results within 25 min, enabling timely initiation of anti-TB therapy [7]. Beyond individual diagnosis, structured implementation of LF-LAM reduces overall TB incidence through three key mechanisms: (1) interrupting transmission by detecting missed paucibacillary cases and halting community and nosocomial transmission chains [8]; (2) minimizing pre-treatment loss-to-follow-up by providing a true “test-and-treat” workflow within a single clinic visit [9]; and (3) supporting the diagnosis of active TB among PLHIV with advanced HIV disease who have signs and symptoms of TB or are seriously ill. Given its limited sensitivity, particularly among PLHIV without AHD, LF-LAM should not be used as a screening or rule-out test for active TB to determine eligibility for Tuberculosis Preventive Treatment (TPT) [10].

At the national level, Thailand represents a high burden setting for both TB and TB/HIV co-infection [1]. Current epidemiological data from the WHO Global Tuberculosis Report 2025 indicate a high national TB incidence rate of approximately 146–155 cases per 100,000 population (>103,000 incident cases annually) [1,11]. Management is further challenged by a high active-treatment mortality rate ranging between 8.7% and 9.8% annually [11]. To mitigate diagnostic delays, the Ministry of Public Health integrated lateral flow urine lipoarabinomannan (LF-LAM) testing into the National Strategic Plan for Tuberculosis Control, Phase 2 (2023–2027), targeting a reduction in national TB incidence to 89 cases per 100,000 population by 2027 [11,12]. This strategy aligns with the Thailand National Guidelines on HIV/AIDS Treatment and Prevention 2021/2022, which endorse LF-LAM as a rapid point-of-care diagnostic to enhance early TB detection among people living with HIV [13].

This rapid, decentralized scale-up of LF-LAM introduces new quality assurance challenges: assay performance relies heavily on precise adherence to procedural timing, prevention of sample contamination, and accurate visual interpretation [13]. Faint bands near the lower limit of detection (Grade 1 intensity) are a recognized source of inter-reader variability, leading to false-positive or false-negative results that compromise clinical decisions [14]. Internal Quality Control (IQC) uses characterized control materials to routinely verify reliable testing performance within a site and is distinct from the LF-LAM assay’s internal control line, which confirms the validity of an individual test. In contrast, External Quality Assessment (EQA), or proficiency testing, independently evaluates performance across sites to identify recurrent errors and guide corrective actions or retraining. Together, IQC and EQA provide complementary mechanisms to ensure reliable and consistent LF-LAM testing across decentralized healthcare settings [15]. Although national EQA systems are well-established in Thailand for conventional TB and HIV assays [16], published data on the development and longitudinal implementation of a national EQA program specifically designed for urinary TB-LAM testing remain scarce.

To address this gap, this study aims to evaluate the development, validation, and longitudinal implementation of the first National EQA program for urinary TB LF-LAM testing among decentralized healthcare facilities in Thailand. Specifically, we assessed participant performance accuracy, characterized recurrent interpretive errors associated with low-intensity positive bands, and identified operational factors influencing testing quality to support the reliable scale-up of LF-LAM testing within the national TB control strategy.

## 2. Materials and Methods

### 2.1. Ethical Considerations

This study was conducted in accordance with the Declaration of Helsinki and approved by the Institutional Review Board of the Department of Medical Sciences (DMSc-EC058). The project was also reviewed in accordance with CDC human research protection procedures and was determined to be non-research. All activities were conducted under a CDC-approved protocol. Patient identifiers and associated personal metadata were entirely anonymized and stripped from the specimens prior to laboratory analysis and EQA panel formulation to ensure absolute data privacy.

### 2.2. Clinical Specimen Sourcing and EQA Panel Preparation

In September 2024 and early 2025, archived frozen human urine specimens with previously confirmed positive or negative TB-LAM results were retrieved to formulate standardized panels for internal quality control (IQC) and external quality assessment (EQA) schemes. De-identified specimens with pre-characterized urinary TB-LAM profiles were obtained from the Clinical Immunology Laboratory, Department of Pathology, Faculty of Medicine Ramathibodi Hospital, Mahidol University, Bangkok, Thailand, which served as the sample provider for this study. As a leading academic medical center laboratory, it specializes in advanced diagnostic immunology, infectious disease serology, and biomarker characterization to support comprehensive patient care and translational research.

These achieved urine specimens were utilized to construct the External Quality Assessment (EQA) and Quality Control (QC) panels as follows:

- Positive Panels: Formulated by a direct single-step 1:32 dilution of strongly reactive TB-LAM-positive urine into an optimized clinical matrix to minimize pipetting variance and ensure high batch-to-batch reproducibility near the targeted diagnostic thresholds.
- Negative Panels: Prepared directly from confirmed TB-LAM-negative urine pools to evaluate network diagnostic specificity and detect false-positive technical artifacts.

To eliminate pre-analytical matrix interference and non-specific background tracking on lateral flow membranes, urine specimens were thawed and clarified by high-speed centrifugation at 10,000× *g* for 5 min at room temperature. To accurately represent the clinical dynamic range, specimens were diluted to target specific semi-quantitative thresholds corresponding to Grades 1–4 against the manufacturer’s reference card, effectively bracketing the assay’s clinical limit of detection from weak-positive to robustly positive levels. The clarified supernatant was aspirated, homogenized, and aliquoted into sterile, low-protein-binding microtubes (80 μL per tube). Finally, the completed aliquots were barcode-blinded, immediately stored at −20 °C under strict temperature monitoring and maintained until distribution for multi-center proficiency testing. During distribution, the EQA and QA panels were transported in a temperature-controlled condition at approximately −20 °C and stored frozen at participating sites if not tested within three days. Stability was confirmed by maintaining the expected LF-LAM results and assigned band intensities throughout the evaluated period.

### 2.3. Sample Characterization and Pre-Qualification Validation

#### 2.3.1. Sample Characterization

The qualitative diagnostic profiles of the prepared panels were verified using a lateral-flow LAM assay (LF-LAM), the commercial Determine™ TB LAM Ag Assay (Abbott Diagnostics Scarborough, Inc., Scarborough, ME, USA). Visual strip readouts were performed under standardized laboratory conditions, with band intensity interpreted against the manufacturer’s reference card to define baseline positive and negative panel status.

#### 2.3.2. Homogeneity Assessment

To evaluate intra-batch uniformity after aliquoting, 10 randomly selected samples, representing 5% of the total prepared batch of 200 tubes, from the production lot were individually tested using the Determine™ TB LAM Ag assay (Abbott Diagnostics Scarborough, Inc., Scarborough, ME, USA). Panel homogeneity was determined based on the qualitative concordance of band reactivity and visual intensity across all replicates prior to field distribution.

#### 2.3.3. Stability Assessment

To assess specimen stability under conditions relevant to the actual distribution process, representative aliquots were stored at −20 °C and longitudinally evaluated at 1-, 2-, and 3-week post-preparation. At each time point, samples were tested using the Determine™ TB LAM Ag assay to monitor for any variations in qualitative reactivity or visual band intensity. For the actual PT distribution, specimens were transported in a temperature-controlled vehicle maintained at approximately −20 °C, with door-to-door delivery directly to participating laboratories. This distribution approach was feasible and no transportation-related issues were encountered.

#### 2.3.4. Long-Term Stability Assessment

Long-term stability was evaluated by storing representative aliquots at −20 °C and testing replicate vials at longitudinal intervals of 1-, 3-, 6-, and 12 months post-preparation. At each interval, samples were analyzed using the Determine™ TB LAM Ag assay to monitor the long-term preservation of qualitative antigen reactivity within the clinical urine matrices.

### 2.4. National Quality Assurance Framework Design and Implementation

#### 2.4.1. Internal Quality Control (IQC) Panel Framework

A standardized TB-LAM internal quality control (IQC) framework was established to verify test kit batch quality and operational performance across participating sites. Each site received one IQC panel containing two well-characterized specimens: one positive control and one negative control. As this was a demonstration project in which TB-LAM test kits were centrally procured and supplied nationwide from a single lot, participating sites were instructed to perform IQC once upon the initial use of the supplied test kit lot, following the instructions provided with the IQC panel. IQC results were submitted through a standardized Google Form to the QA provider for evaluation within the specified reporting period. Panel distribution scaled across testing cycles, expanding from 7 participating laboratories in IQC Round 1 (June 2025) to 68 laboratories in IQC Round 2 (January 2026).

#### 2.4.2. External Quality Assessment (EQA) Program

A prospective, multicenter national EQA program was conducted across four sequential rounds between March 2025 and April 2026, running concurrently with the IQC cycles. A subset of participating IQC laboratories joined the EQA program, with all 30 laboratories from Round 1 re-evaluated in Round 2, specifically ensuring the re-inclusion of sites that failed the initial round. Throughout all implementation cycles, each round evaluated analytical procedures, diagnostic accuracy, and interpretation proficiency using a longitudinal, blinded panel of three characterized positive and negative specimens distributed to participating medical laboratories.

### 2.5. Participant Testing and Data Collection

Blinded EQA panels were distributed frozen in insulated, leak-proof containers via temperature-controlled transport maintained at approximately −20 °C to participating Thai healthcare facilities (comprising both government facilities under the Ministry of Public Health and private healthcare facilities across regional, provincial, and district levels). Upon receipt, participants tested the panels within 1 week under routine conditions using the Determine™ TB LAM Ag assay by applying 60 μL of sample and interpreting results visually at 25 min against the manufacturer’s reference card. Both qualitative results (Positive/Negative) and semi-quantitative band intensities (Grades 1–4) were recorded. All data—including test result photographs submitted as supportive evidence for visual verification, captured under standardized lighting conditions and within the precise reaction reading window—were submitted within a 14-day window via a secure web platform.

### 2.6. Performance Evaluation and Corrective Actions

#### 2.6.1. Scoring and Proficiency Classification

Diagnostic proficiency was evaluated at the participating laboratory using a standardized scoring system managed by the Department of Medical Sciences. One designated tester performed the testing at each site, and performance for each specimen was classified based on qualitative concordance with predefined reference results as follows:

Satisfactory (Total Proficiency): Defined as 100% qualitative concordance within a round, corresponding to correct interpretations for all specimens (2/2 in QC rounds; 4/4 in EQA rounds). Minor variations in visual band grading (Grades 1–4) for true-positive specimens were deemed acceptable provided the final qualitative interpretation remained positive.

Unsatisfactory (Suboptimal Proficiency): Any discordance between the participant’s reported qualitative result and the reference value was classified as unsatisfactory.

#### 2.6.2. Root-Cause Analysis and Remedial Interventions

Facilities with unsatisfactory performance underwent a structured corrective action protocol. Participants completed a standardized troubleshooting checklist to investigate potential procedural, operational, or environmental causes of error.

### 2.7. Statistical Analysis

Statistical analyses were performed using the IBM SPSS Statistics 22.0 program. Participation compliance, reporting rates, and laboratory passing proficiencies across sequential rounds were summarized using descriptive statistics, including frequencies and percentages. To assess chronological training and performance progressions, changes in diagnostic failure modes (false-positive and false-negative incident rates) across consecutive EQA cycles were analyzed using Fisher’s exact test. Statistical significance for all trend evaluations was defined at *p* < 0.05.

Missing Data Handling: Non-reporting sites were excluded from diagnostic concordance denominators using a per-round complete-case approach. Characterization of Low-Intensity Band Errors: We added a detailed framework explaining how Grade 1/trace-band results were stratified into distinct misclassification modes (false-negatives vs. false-positives) and evaluated for root-cause operational factors (such as timing non-compliance and reader experience) using logistic regression.

## 3. Results

### 3.1. EQA Panel Validations, Homogeneity, and Stability Profiles

**Figure 1:**
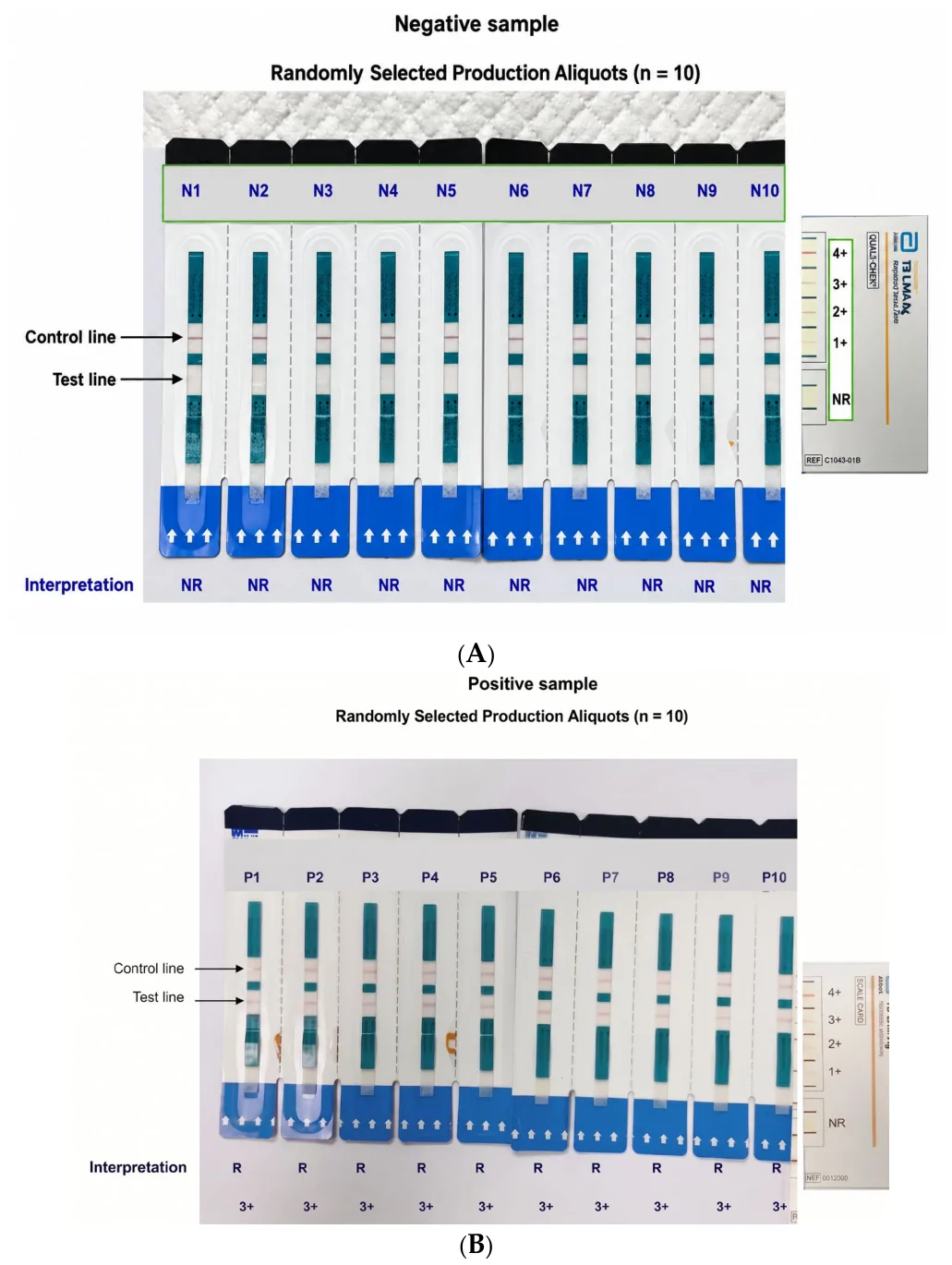
Visual comparison of 10 randomly selected aliquots from (**A**) negative panels (N1–N10) and (**B**) positive panels (P1–P10), demonstrating consistent intra-batch qualitative homogeneity at the target diagnostic threshold.

The TB lateral flow urine lipoarabinomannan (LF-LAM) EQA panel validation phase confirmed successful characterization, preparation, and standardization of the clinical urine matrices. Pre-qualification validation demonstrated that panel antigen concentrations successfully spanned the assay’s dynamic operating range, targeting semi-quantitative categories corresponding to Grades 1–4 against the manufacturer’s reference card to bracket the clinical limit of detection. Homogeneity assessment of 10 randomly selected aliquots from the production lot demonstrated 100% concordance in qualitative immunochromatographic band reactivity and intensity (Table 1, Figure 1). No intra-batch variation was observed, confirming that the specimen processing and serialization protocols yielded adequately uniform panel materials.

**Figure 2:**
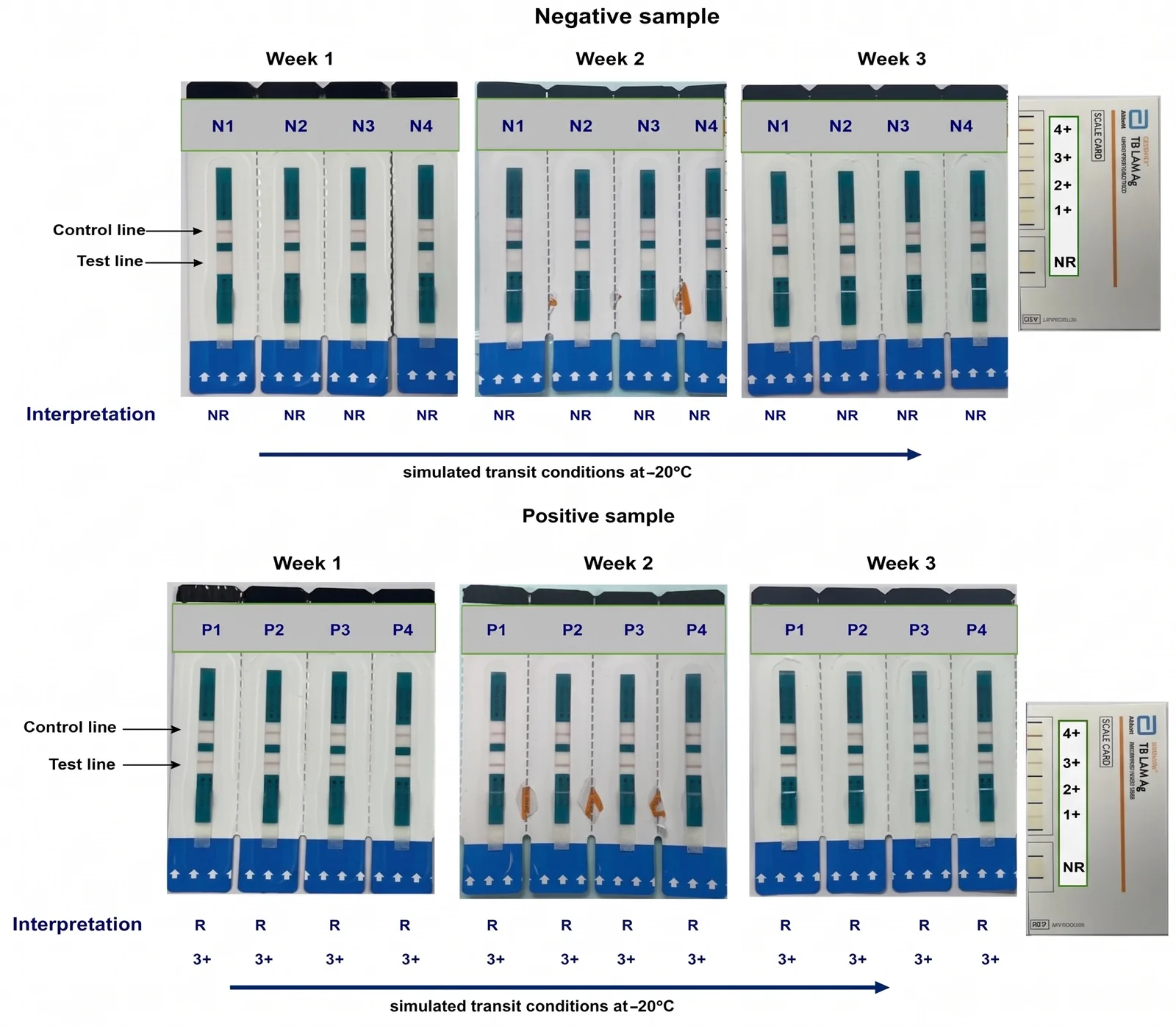
Visual reactivity of TB LF-LAM validation under simulated transportation conditions at −20 °C over 3 weeks (Weeks 1, 2, and 3). Quadruplicate validation strips (*n* = 4) demonstrated consistent reactivity across the testing period. Abbreviations: NR, non-reactive; R, reactive.

**Figure 3:**
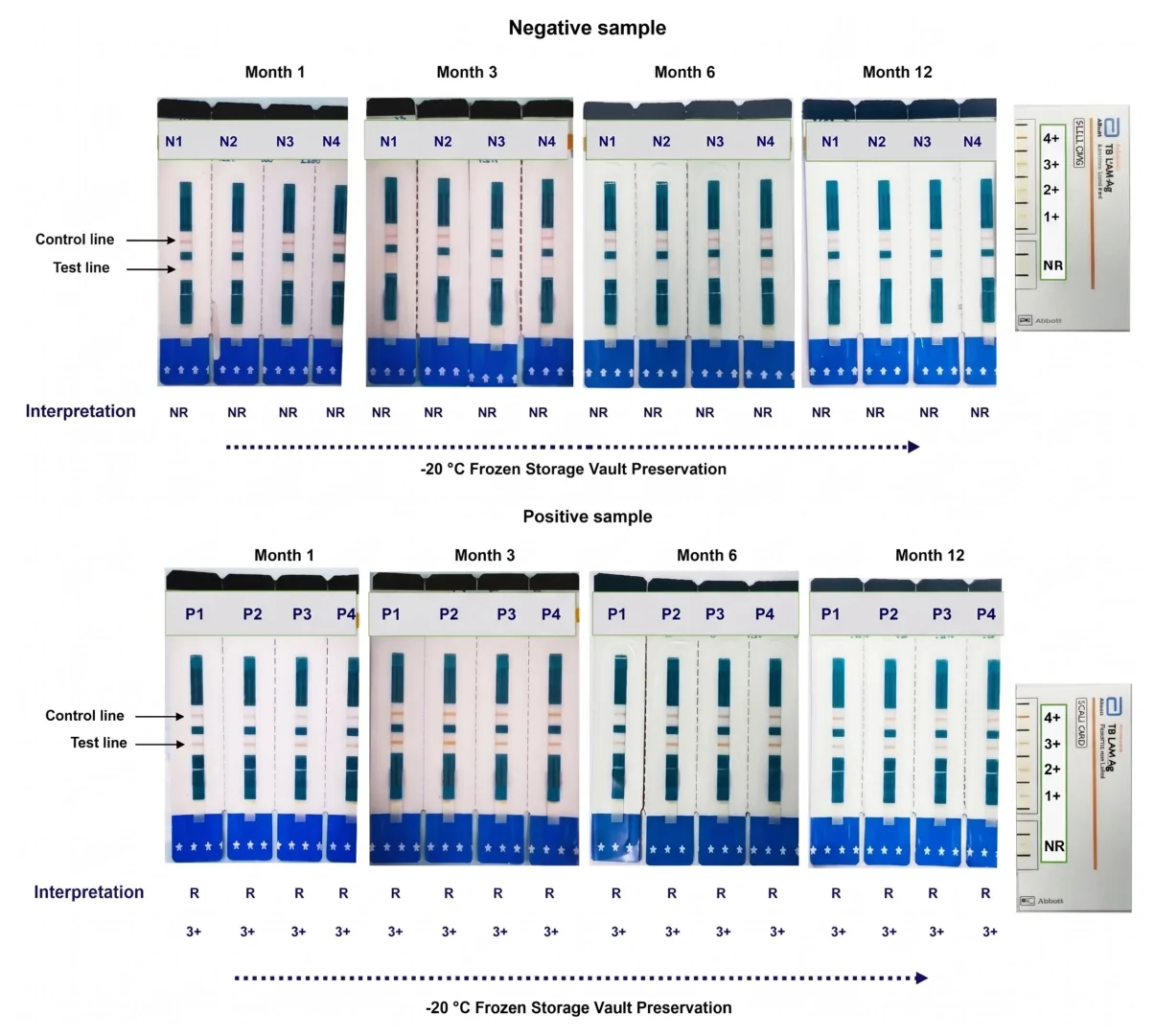
Visual reactivity of TB LF-LAM panels during long-term stability testing at −20 °C over 12 months (Months 1, 3, 6, and 12). Triplicate validation strips for negative (*n* = 4) and positive (*n* = 4) samples demonstrate consistent reactivity and band intensity across the testing period. Abbreviations: NR, non-reactive; R, reactive.

In the transportation stability assessment, the TB LF-LAM remained stable under simulated transit conditions at −20 °C. Evaluations at 1-, 2-, and 3-week post-preparation showed no change in visual band scores and diagnostic outcomes (Table 2, Figure 2). Long-term stability tracking at −20 °C showed no evidence of antigen degradation or a reduction in band intensity at 1-, 3-, 6-, and 12-month time points (Table 3, Figure 3). These findings indicate that the panels were sufficiently stable for 3-week transport and 12-month long-term storage at −20 °C.

### 3.2. Performance of the Internal Quality Control (IQC) Program

During the initial program establishment phase, spanning distribution rounds in June 2025 and January 2026, internal QC panels comprising paired positive and negative controls were distributed to participating laboratories. The network coverage expanded significantly, with the number of participating laboratories expanding from 7 participating laboratories in IQC Round 1 (June 2025) to 68 laboratories in IQC Round 2 (January 2026). The program achieved high compliance, with round-specific response rates ranging from 89.7% to 100% and passing rates ranging from 85.7% to 100%, reflecting early variations in technical familiarity with the urinary antigen matrix. Detailed QC performance across the validation cycles is presented in Table 4.

Qualitative error tracking identified a distinct pattern of analytical anomalies. Diagnostic failures recorded during QC Round 1 consisted exclusively of false-positive results. Root-cause analysis revealed that this discordance was primarily driven by the misinterpretation of faint bands below Grade 1 intensity on the immunochromatographic membranes, as well as noncompliance with the manufacturer’s reference scale-card interpretation instructions.

### 3.3. National External Quality Assessment (EQA) Program Performance

Evaluation of the longitudinal EQA data demonstrated a notable positive transition in network diagnostic proficiency and error control over time (Table 5). Although total site participation shifted across implementation cycles (30 facilities in 2025 versus 64 and 42 facilities across subsequent rounds in 2026) due to phased national integration, a stratified comparative analysis ruled out selection bias. Specifically, baseline pass rates between newly onboarded sites and core repeat-participating laboratories showed no statistically significant differences (*p* > 0.05, Fisher’s exact test). Longitudinal tracking of repeat participating sites revealed distinct individual facility learning curves—characterized by a progressive reduction in scoring errors over successive rounds—while system-wide improvements reflected broader standardization and technical support across all participating health tiers.

- Significant Improvement in Passing Rates: The overall laboratory passing rate significantly increased from 83.3% (25/30) in Round 1/2025 to 97.5% (39/40) by the final Round 2/2026. Fisher’s exact test comparison of proficiency between the first and final rounds confirmed that this upward trajectory in network performance was statistically significant (*p* = 0.046), reflecting a sharp reduction in cumulative laboratory failures as the program progressed.
- Significant Reduction in False-Positive Incidents: The network witnessed a distinct downward trend in false-positive interpretations, which dropped sequentially from 5 incidents in Round 1/2025 to 0% (0/40) in Round 2/2026. This chronological reduction in user-driven false-positive errors was statistically meaningful (*p* < 0.05), validating the efficacy of targeted corrective training and the distribution of reference match-cards in minimizing operator misinterpretation of trace background lines.
- Analysis of False-Negative Emergence: In contrast to the false-positive trend, a single false-negative incident (2.5%) emerged for the first time in Round 2/2026. However, statistical analysis indicated that this isolated occurrence did not represent a significant increase in the network’s false-negative rate (*p* > 0.05), confirming it as an isolated pre-analytical or procedural error at a single peripheral site.

## 4. Discussion

Establishing a rigorous EQA program for urine LAM is essential to ensure high diagnostic accuracy, directly minimizing false-positive and false-negative results so that vulnerable patients diagnosed with TB receive timely, accurate TB treatment.

The implementation of Thailand’s first national External Quality Assessment (EQA) program for urinary TB-LAM assays demonstrates that centralized proficiency testing can significantly enhance laboratory performance across decentralized point-of-care networks [15,16]. The progressive increase in passing rates—from 83.3% in Round 1/2025 to 97.5% by Round 2/2026—indicates that longitudinal external assessments and structured corrective feedback successfully standardized immunochromatographic assay interpretation [14]. This structural approach is critical for lateral flow assays, which are highly vulnerable to operator-dependent subjectivity and benefit immensely from continuous quality assurance frameworks [15,16].

Root-cause analysis during the early quality control (QC) and EQA phases revealed that diagnostic failures were predominantly driven by false-positive misinterpretations of baseline-negative specimens [14]. These misinterpretations highlight the technical challenges in visually reading faint bands near the detection threshold and emphasize the critical need for strict adherence to standardized reading protocols [14,15].

The complete elimination of false-positive results by the final round suggests that the distribution of reference materials, protocol reinforcement, and targeted retraining were highly effective [13]. Conversely, the detection of a single false-negative result in the final round highlights the utility of ongoing proficiency monitoring and prompt corrective actions [16]. While rare, false-negative findings warrant continued oversight to prevent potential diagnostic delays in high-risk individuals.

Although infrequent, false-negative errors cause serious clinical consequences by delaying life-saving treatment initiation in vulnerable patients with advanced HIV disease (AHD) [2,9]. Overall, these findings reinforce the value of a longitudinal EQA system over standalone, one-time training initiatives [16].

### 4.1. Comparative Analysis with Global EQA Implementations

The baseline vulnerabilities identified in Thailand’s network align with operational challenges documented during decentralized LF-LAM scale-up efforts in other high TB/HIV burden settings, such as inter-operator variability and difficulty reading faint Grade 1 bands under suboptimal lighting [14,15]. However, while some international programs responded to these challenges by raising the diagnostic rule-in threshold to Grade 2 to mitigate operator error [14,15], Thailand maintained the WHO-recommended Grade 1 threshold [5,12]. By combining this high-sensitivity cutoff with the strategic distribution of standardized reference match-cards and targeted retraining, the national program successfully minimized interpretation errors without sacrificing clinical sensitivity [14].

Furthermore, unlike quality assurance strategies that rely on sporadic, on-site supervisory checklists, Thailand established an active, longitudinal proficiency-testing network utilizing highly stable clinical specimens [13]. This centralized approach likely underpinned the network’s high reporting compliance (95.2%) and final proficiency rate (97.5%), outperforming decentralized testing networks that lack robust electronic verification and centralized data management [16,17]. Furthermore, these findings strongly advocate for incorporating a permanent, standardized urine LAM EQA scheme into the national implementation framework to maintain long-term testing quality and program sustainability.

### 4.2. Multi-Tiered Benefits of the Centralized Quality Assurance Ecosystem

The successful establishment of the national TB-LAM quality framework yields critical implications for clinical care, laboratory capacity, and health-system efficiency [2,12]. Mechanistically, reducing the network false-positive rate to 0% eliminated the risk of inappropriate anti-TB therapy and associated drug toxicities in vulnerable PLHIV [2,8], while the rapid remediation of the single false-negative event preserved the assay’s rapid rule-in utility for critically ill inpatients [7,9]. Concurrently, the upward proficiency trajectory indicates meaningful local capacity building [14]. Repeated root-cause investigations have strengthened frontline quality awareness and fostered a sustainable culture of internal auditing, driving peripheral laboratories closer to ISO 15189-oriented operational standards [16].

At the macro-system level, a centralized data repository functions as an early warning system capable of detecting lot-specific manufacturing defects or transport-induced reagent degradation prior to widespread clinical impact [14,16]. Additionally, standardizing point-of-care readouts minimizes diagnostic waste, refines procurement forecasting, and enhances the return on investment in national laboratory infrastructure [12,16]. By reducing invalid runs caused by procedural errors—such as incorrect sample volume dispensing or premature readout—the program ensures optimal kit utilization [14]. More broadly, this robust quality ecosystem supports the early, accurate detection of active TB among PLHIV with AHD, catalyzes timely treatment initiation to reduce mortality, and offers a scalable quality assurance model for other high-burden countries globally [1,5,9].

### 4.3. Study Limitations and Future Directions

Five limitations of this study must be acknowledged. First, the limited sample size of participating facilities and EQA rounds restricts the statistical power and precision of subgroup comparisons across different healthcare settings. Second, the EQA panels were prepared using centrifuged, pre-characterized clinical specimens sourced from a single tertiary care center and diluted to targeted thresholds [14]. While this approach optimized sample homogeneity and ensured a 12-month shelf life at −20 °C, it may not fully capture the complex pre-analytical variability encountered in routine field settings; there, uncentrifuged urine containing turbidity, amorphous crystals, mucous, or hematuria could interfere with lateral flow membrane migration [14,15]. Future national EQA cycles should investigate preparation methods that better incorporate these challenging matrix features while preserving antigen stability [14]. Third, this evaluation was restricted exclusively to the single commercial LF-LAM test kit brand that possessed Thailand FDA approval and was in routine clinical use during the study period; consequently, these findings may require re-validation if alternative diagnostic platforms are introduced [14]. Fourth, although the web-based reporting system successfully captured longitudinal data across decentralized facilities, scaling this digital data collection framework to remote, resource-limited community health posts remains a practical infrastructure challenge [16,17]. Fifth, while transportation at −20 °C proved reliable and maintained specimen integrity throughout this pilot, exploring alternative formats such as liquid-stable or freeze-dried quality control materials represents a vital direction for future program optimization; these formulations could eliminate strict cold-chain requirements, thereby simplifying logistics, reducing operational costs, and improving accessibility for peripheral district and community hospitals with limited infrastructure.

## 5. Conclusions

The successful establishment of Thailand’s first national EQA program for urinary TB-LAM testing confirms that a centralized proficiency testing framework can effectively standardize point-of-care diagnostics across decentralized clinical tiers. Over a two-year longitudinal evaluation, the national quality framework drove a statistically significant reduction in network failure rates and successfully eliminated operator-driven false-positive errors through targeted retraining and the implementation of high-resolution reference visual match-cards. This continuous quality ecosystem not only ensures high diagnostic specificity at frontline clinics but also builds long-term institutional quality awareness, moving decentralized laboratories closer to international standard compliance. Ultimately, this scalable external quality model may inform other high TB/HIV burden nations seeking to reinforce point-of-care testing infrastructure, minimize diagnostic kit waste, and scale up life-saving preventive and therapeutic anti-TB interventions.

## Figures and Tables

**Table 1:** Homogeneity validation metrics for TB LF-LAM EQA panel.

Samples/Number of Replicates (*n*)	Test Results of Determine™ TB LAM Ag Assay				Homogeneity Validation
	Control Line	Test Line	Interpretation	Consensus Diagnostic Accuracy	Lot Uniformity
Negative (*n* = 10)	+	0	Negative	100% agreement	Adequately Homogeneous
Positive (*n* = 10)	+	3+	Positive	100% agreement	Adequately Homogeneous

0 indicates no band; ‘+’ shows the band is present; ‘3+’ reflects the strength of the band; the 3+ intensity is determined by comparing it to the scale card.

**Table 2:** Reactivity validation of TB LF-LAM panels under simulated transportation at −20 °C.

Sample	Tube Number	Day 0 (Baseline)	1-Week Post-Preparation	2-Week Post-Preparation	3-Week Post-Preparation	Consensus Diagnostic Accuracy	Stability Status
		9 October 2024	17 October 2024	24 October 2024	31 October 2024		
Negative	N1	0	0	0	0	100%	Stable/ Transport-Ready
	N2	0	0	0	0	100%	Stable/ Transport-Ready
	N3	0	0	0	0	100%	Stable/ Transport-Ready
	N4	0	0	0	0	100%	Stable/ Transport-Ready
Positive	P1	3+	3+	3+	3+	100%	Stable/ Transport-Ready
	P2	3+	3+	3+	3+	100%	Stable/ Transport-Ready
	P3	3+	3+	3+	3+	100%	Stable/ Transport-Ready
	P4	3+	3+	3+	3+	100%	Stable/ Transport-Ready

0 indicates no band; ‘3+’ reflects the strength of the band; the 3+ intensity is determined by comparing it to the scale card.

**Table 3:** Long-term stability of TB LF-LAM panel reactivity at −20 °C over 12 months.

Samples	Tube Number	Month 1 Post-Archiving	Month 3 Post-Archiving	Month 6 Post-Archiving	Month 12 Post-Archiving	Consensus Diagnostic Accuracy	Long-Term Stability
		8 November 2024	9 January 2025	9 April 2025	16 October 2025		
Negative	N1	0	0	0	0	100%	Stable/Preserved
	N2	0	0	0	0	100%	Stable/Preserved
	N3	0	0	0	0	100%	Stable/Preserved
	N4	0	0	0	0	100%	Stable/Preserved
Positive	P1	3+	3+	3+	3+	100%	Stable/Preserved
	P2	3+	3+	3+	3+	100%	Stable/Preserved
	P3	3+	3+	3+	3+	100%	Stable/Preserved
	P4	3+	3+	3+	3+	100%	Stable/Preserved

0 indicates no band; ‘3+’ reflects the strength of the band; the 3+ intensity is determined by comparing it to the scale card.

**Table 4:** Operational response metrics and diagnostic passing rates for the TB-LAM IQC program, 2025–2026.

Operational Round (year)	Number of Dispatched Sites (*N*)	Completed Responses (*n*, %)	Laboratories Achieving Passing Status (*n*, %)	Error Identified/ Cause of Error
QC Round 1 (2025)	7	7 (100.0%)	6 (85.7%)	False Positive/ Visual misreading *
QC Round 2 (2026)	68	61 (89.7%)	61 (100.0%)	None/ Perfect Consensus

* Visual misreading: misinterpretation of faint bands below Grade 1 intensity on the immunochromatographic membrane, resulting in discordance with the expected LF-LAM result.

**Table 5:** Longitudinal proficiency trends and error distribution across the four national TB-LAM EQA rounds, 2025–2026.

EQA Round/Year	Sites Enrolled (*N*)	Reporting Rate (*n*, %)	Passing Rate (*n*, %)	False-Positive Incidents (*n*)	False-Negative Incidents (*n*)
Round 1/2025	30	30 (100%)	25 (83.3%)	5	0
Round 2/2025	30	29 (96.7%)	27 (93.1%)	2	0
Round 1/2026	64	62 (96.9%)	61 (98.4%)	1	0
Round 2/2026	42	40 (95.2%)	39 (97.5%)	0	1
*p*-value ^1^	—	0.490	0.046 *	0.021 *	0.571

Notes: ^1^ Statistical significance was evaluated using Fisher’s exact test to compare proportions between the baseline cycle (Round 1/2025) and the final cycle (Round 2/2026). Statistically significant differences (*p* < 0.05) are highlighted in bold with an asterisk (*).

## Data Availability

The aggregated consensus data and laboratory proficiency metrics supporting the findings of this study are available within the article matrices. Individual laboratory raw performance datasets are available upon reasonable request from the corresponding author due to national public health data privacy and institutional security regulations.
